# The Experience of Insomnia in Patients With Schizophrenic Disorder: A Qualitative Study

**DOI:** 10.3389/fpsyt.2021.805601

**Published:** 2022-01-17

**Authors:** David Batalla-Martín, Maria-Antonia Martorell-Poveda, Angel Belzunegui-Eraso, Eva Miralles Garijo, Ana Del-Cuerpo Serratosa, JuanCarlos Valdearcos Pérez, Miquel Montané Escobar, Marina Lopez-Ruiz

**Affiliations:** ^1^Nou Barris Mental Health Center, Barcelona, Spain; ^2^Nursing Department, Faculty of Nursing, Rovira i Virgili University, Tarragona, Spain; ^3^Chair for Social Inclusion, Rovira i Virgili University, Tarragona, Spain; ^4^Service of Psychiatry and Psychology, HM-Sant Jordi Clinic, Barcelona, Spain

**Keywords:** insomnia, schizophrenia, sleep disturbance, quality of life, qualitative research, illness experience

## Abstract

**Background:**

Insomnia is a health problem that particularly affects people with schizophrenia. Its repercussions go beyond the disorder itself and affect many areas of life. The aim of the present study is to explore the clinical symptoms and consequences of insomnia in patients diagnosed with schizophrenic disorder and the perceptions of these patients regarding the care they receive.

**Methods:**

The study takes a qualitative approach and uses semi-structured interviews to conduct a descriptive and interpretive analysis of 3 clinically different clusters of patients. These 3 clusters have been defined by using two-step cluster analysis based on the results of the ISI (Insomnia Severity Index) and EQ-5D scales (EuroQol-5D) and the presence of certain diagnostic symptoms in a sample of 170 patients. The final sample was 31 subjects. The analysis was based on a hermeneutic analysis of the patients' narratives regarding their experiences of insomnia.

**Results:**

The patients' narratives show differences in the intensity and experience of insomnia depending on the severity, as well as its impact on their quality of life. Insomnia has a huge emotional impact. Participants describe ruminations and obsessive thoughts as a key factor hindering falling asleep. Some of the everyday actions they perform encourage the chronicity of insomnia. The desired health response must include interventions that are effective, such as cognitive-behavioural therapy, and powerful, such as pharmacological solutions. Psychoeducation and advice on sleep hygiene are highly valued tools as preventive strategies.

**Conclusions:**

To know the experience of users gives us a more comprehensive understanding of insomnia complexities and brings some new intervention strategies in patients with mental disorders. It is important that health professionals intervene preventively to stop the disorder from becoming chronic.

## Introduction

Sleep is an temporary unconscious physiological state in which bodily functions and mental activities undergo changes that are of great importance for mental and physical balance (1). The need for sleep is biological and appears regularly in cycles in order to ensure that the body rests and regenerates the energy spent. This phenomenon occupies one third of human life (2).

Sleep disorders have high prevalence rates in the population with mental disorders. Cohrs et al. (3) describe that disturbed sleep is present in between 30 and 80% of the population with schizophrenia, although they do not specify the type of sleep disorder. Kaufman et al. (4) also place sleep problems in more than 70% of the population with a mental disorder. The most common sleep disorder in the population with a mental disorder is insomnia (5). According to Mondal et al. (6), 73.4% of psychiatric patients have this sleep disorder, where patients with schizophrenia show a prevalence of 23.8%. This figure is similar to that observed by Hou et al. (7) and Batalla-Martin et al. (8), who found the prevalence at 28.9 and 23.2%. Mondal et al. also states that the diagnostic symptoms of insomnia show a prevalence of 78.2% (6).

Insomnia is, on many occasions, treated as a symptom of others mental disorders (9), however, some evidence suggest that insomnia may precede these mental disorders (10). In a consensual opinion made at the National Institutes of Health (11) conference they agreed insomnia should be considered as a primary disease rather than secondary. Insomnia, considering ICD-10 criteria, is defined by difficulties with sleep initiation or maintenance, early awakening, non-restorative sleep; repercussions on wakefulness, such as concern, tiredness, decreased function. The primary insomnia needs the absence of a cause-effect relationship between insomnia and most of the other conditions (10).

The impact of insomnia goes beyond the disorder itself and directly affects people's health (12) and quality of life (13). It is almost always associated with daytime fatigue and mood swings, including irritability, dysphoria, tension, helplessness, and depressive moods (14). Some studies even suggest that untreated chronic insomnia may be one of the risk factors for developing major depression (15, 16). The study of bidirectionality between insomnia and mental disorders, specifically between insomnia and depression and anxiety have shown that insomnia is associated with a major risk of disruption, being insomnia a prodromal predictor (17, 18). Also insomnia and sleep alterations can predict acute exacerbation of psychotic symptoms (19, 20). Nevertheless, the links between sleep disorders and schizophrenia have not been examined in depth, in part because there are numerous factors that contribute to their comorbidity, such as medication (20). Patients with insomnia often have somatic complaints, typically gastrointestinal and respiratory problems, headaches, and non-specific pain (11).

Sleep quality and quality of life have been shown to have a close relationship. Zeitlhofer et al. (21) found that sleep quality could be a good predictor of poor quality of life. The impact of insomnia on health-related quality of life (HRQoL) is significant (13, 22) and directly affects the ability to perform everyday activities or participate in social activities (23), two important areas that are already affected by the underlying disorder in patients with schizophrenic disorder.

Beyond the clinical symptoms themselves, insomnia also affects other areas of the lives of people who suffer from it. This is why, in addition to a quantitative analysis, studies need to focus both culturally and socially on the actual narrative of the people affected. Such an approach makes it possible to understand more clearly the experience of this disorder and its implications, by focusing on the *illness* (psychobiological dimension) and *sickness* (sociocultural dimension) without neglecting the *disease* dimension (biological dimension) (24). As Byron Good rightly states: health and disease do not happen in the body, but in life (25).

Although most studies have analysed insomnia quantitatively, in their systematic reviews Araújo et al. (26) and Waite et al. (27) have found that the literature on qualitative analysis has doubled in the last 5 years and have concluded that qualitative approaches in the medical field of behavioural sleep, and more specifically, insomnia disorder, could provide a more comprehensive understanding of the phenomenon and its complexities.

Such qualitative studies have focused on analysing the experience of insomnia mainly in the general population (28–34), the types of treatments such populations receive and their expectations (35–39), and the knowledge of professionals regarding the provision of care for people with insomnia (35, 36, 40–42). However, in comparison, very few articles have analysed the narratives of patients with schizophrenic disorder who suffer from insomnia (38, 39, 43, 44), and there are no studies in the Spanish population. Authors who have studied insomnia in these people have highlighted the importance of understanding the pathology in terms of the patient's own perceptions and experience in order to be able to adapt future interventions to treat insomnia (38, 45).

The different qualitative studies and the narratives themselves have generally been studied using two methodological techniques, that is, the semi-structured interview and focus groups. These have given rise to analysis units normally centred on sleep problems; causes of sleep problems (belief that sleep problems cannot change, trauma and adversity, lifestyles and lack of motivation, effects of treatments), the impacts of sleep problems; medicalization; and possible treatments (39, 45).

The aim of this study is to qualitatively explore the experience of insomnia, its clinical symptoms, its repercussions and the perception of the care received in patients diagnosed with schizophrenic disorder who are monitored and treated, as outpatients, at the Mental Health Center of Nou Barris, Barcelona, with the intention of adapting future interventions to their clinical, psychological and social realities. In the Methodology section we explain the method for creating different clusters depending on the severity of the insomnia, the selection of the informants, and the data collection and data analysis processes.

## Methods

### Design and Procedure

The study was based on a hermeneutic analysis of the narratives regarding the experience of insomnia in patients diagnosed with schizophrenic disorder.

Two data analysis processes were performed. First, the analysis clusters were created, designed using the Two Step (46) statistical process with the statistical program SPSS v.20, based on the results obtained in the previous prevalence study (8). Subsequently, to validate and corroborate the results obtained, a hierarchical cluster analysis was performed using the Ward method. Finally, to contrast these two processes, a statistical analysis (Cohen's Kappa coefficient) was performed to ensure agreement between the two procedures as a validation of the Two Step procedure.

In the previous study, Insomnia in Schizophrenia Patients: Prevalence and Quality of Life (8), in which we determined the prevalence of insomnia in patients suffering schizophrenia at an outpatient mental health centre in Barcelona (Catalonia, Spain), we interviewed 267 participants, from whom we selected all those participants who presented at least one of the diagnostic criteria for insomnia according to the International Statistical Classification of Diseases and Related Health Problems (ICD-10) The presence of insomnia was evaluated by means of the Oviedo Sleep Questionnaire (OSQ) (47) (Supplementary Figure 1). Specifically, we selected 170 patients to form the sample on which we performed the cluster analysis for the present study.

The variables used to formalise the clusters were: the presence of clinical symptoms of insomnia according to the ICD-10 criteria (dichotomous variable), the ISI scale result (ordinal variable) and the EQ-5D visual scale result (continuous variable). The variables of the elaboration of the clusters were selected for their relevance in the insomnia experience. The first variable, presence of insomnia or the presence of symptoms, was chosen according to the criteria of the Oviedo Sleep Questionnaire to select the sample. The variable severity of insomnia, assessed with the ISI scale, was chosen to be associated with the severity of symptoms and sleep quality (48), in addition, it assesses patients' perception and subjective dimensions of insomnia (49). Finally, the health-related quality of life variable is chosen to be associated with the same severity of insomnia, productivity and fatigue (50). In addition, it is related in patients with schizophrenia to poor sleep quality (51).

The resulting three clusters were named: Severe-Moderate Insomnia; Mild Insomnia; No insomnia with symptoms. The term “cluster” refers only to the statistical concept of the procedure and not to a stratified participant selection process. Carrying out this procedure provides greater coherence and internal consistency and ensures statistical differentiation of the sample.

Second, once the analysis groups had been created, semi-structured interviews were carried out with the patients in each of the clusters until concept saturation was reached for each cluster. The informants were selected in a non-probabilistic consecutive way and were offered the possibility to participate in a follow-up visit with the same professional. The interviews were conducted by the principal investigator in individual sessions at the mental health centre. Participating patients were assigned an anonymous code to conceal their identity during the analysis process. This code consisted of the abbreviation PAC (derived from the Catalan word “pacient,” meaning patient) followed by the number of the interviewee (X), then a full stop and then the number of the cluster to which the patient belonged (Y): PACX.Y.

The study met the rigorous international ethical recommendations for medical research according to the Declaration of Helsinki. Participants were informed of their rights and that the data provided would be treated confidentially in accordance with (EU) Regulation No. 2016/679 and Organic Law 3/2018 of 5 December on the Protection of Personal Data and the guarantee of digital rights. The study was approved by the Ethics Committee of the Union of Catalan Hospitals (UCH) with the registration code CEI 20/22.

### Subjects

The sample included 31 patients, needed to ensure saturation of concepts, out of a total of 170 potential participants in the previous study (8). Subjects were selected in a non-probabilistic consecutive way and the inclusion criteria were that they should: be men and women of legal age diagnosed with schizophrenia, present any of the diagnostic symptoms of insomnia according to the ICD-10 criteria and be included in one of the analysis clusters, be a patient of the Mental Health Centre and have an intact literacy ability. Exclusion criteria were: being unable to speak either Catalan or Spanish, being diagnosed with learning disabilities, having neurological disorders with cognitive impairment or acute decompensation of the psychiatric disorder.

The selection of the informants will be made through the elaboration of the clusters.

### Instruments

To create the clusters, a cluster analysis was performed using the participants' answers to two questionnaires: the Questionnaire of the Insomnia Severity Index (ISI) and the EquoQol-5D scale (EQ-5D).

The presence of insomnia was assessed using the Oviedo Sleep Questionnaire (52). Instrument designed to aid in the diagnosis of insomnia-type sleep disorders based on the criteria of ICD-10. Paz García-Portilla et al. (47) examined the reliability and validity of the OSQ in patients with severe mental disorders, with an internal consistency for the items making up the insomnia scale of 0.91. The consistency value for the total OSQ was 0.90. In patients with schizophrenia, the OSQ has therefore been shown to have good psychometric performance.

The OSQ is a brief semi-structured interview that allows us to take an exhaustive clinical history of the patients' sleep-wake rhythms. The OSQ consists of 9 items (OSQ21 to OSQ24, OSQ3 to OSQ7) that evaluate the nature of the insomnia (difficulties with sleep initiation or maintenance, early awakening, non-restorative sleep), its repercussions on wakefulness (concern, tiredness, decreased function) and its severity. Items OSQ21 to OSQ24 together with OSQ7 constitute the algorithm for categorical insomnia diagnosis by the ICD-10 criteria. The ICD-10 diagnostic algorithm for insomnia is as follows:

- At least one of the four items from OSQ21 to OSQ24 must appear a minimum of 3 days a week (difficulties with initiating or maintaining sleep, reaching restorative sleep or waking up at the normal hour), resulting in a score of ≥3. And also, item OSQ7 must be present at least 3 days a week (concern, tiredness or decreased function due to night-time sleep disorders), resulting in a score ≥ 3.

The variable severity of insomnia was obtained through the questionnaire, *Insomnia Severity Index* (ISI) (53), an instrument that briefly assesses the severity of insomnia in the general population according to the DSM IV and the international classification of sleep disorder (ICSD) criteria. This tool has shown appropriate psychometric properties in the English version, with a reliability of internal consistency values (Cronbach α) of between 0.74 and 0.90, and test-retest reliability equal to 0.89 in the month of the evaluation, 0.77 after 2 months and 0.73 after three (54, 55). It has been validated in Spanish obtaining optimal results for use in this population, where it showed an internal consistency of 0.82 (56).

The variable health-related quality of life (HRQOL) was obtained according to the EuroQol-5D Scale (EQ-5D) (57), specifically through the visual analogue scale (EQ-VAS) of the same tool, which has a test-retest reliability of between 0.86 and 0.90 (58). This scale has also been validated in Spain and in Spanish (59), showing valid psychometric properties even in people with schizophrenic disorder (60).

### Data Collection

The narratives of patients with schizophrenia who participated in the study of insomnia were obtained through semi-structured interviews that were conducted and recorded in the participant's commonly used language (Catalan and/or Spanish).

The interview questions were adapted from Arthur Kleinman's approach (61) to the cultural aspect of intercultural medical evaluations, which proposes the following eight questions: (1) What do you think has caused your problem? (2) Why do you think it started when it did? (3) What do you think your problem does inside your body? (4) How severe is your problem? Will it have a short or long course? (5) What kind of treatment do you think you should receive? (6) What are the most important results you hope to receive from this treatment? (7) What are the chief problems your illness has caused you? (8) What do you fear most about your illness/treatment?

Other studies have used Kleinman's eight questions to determine the experience of patients with different disorders. Metta (62) used them to study the experience of patients with diabetes, whereas Henry (33) used them to study insomnia in the general population. In our research, these 8 questions are used to try to understand the insomnia experience in patients with schizophrenic disorder.

We also added two more questions to the interview: 0) What does sleep mean to you? And 9) How do you feel about participating in a group intervention to address your sleep problem? All informants knew the aim of the study at the time that the interview was done.

The interviews were recorded between May and September 2020. A Sony ICD-PX370 voice recorder was used for the recording. All interviews were conducted in the nursing office of the Ambulatory Mental Health Centre in Nou Barris, Barcelona, Catalonia, Spain.

A sociodemographic data questionnaire was also given to all study participants to more accurately determine the characteristics of the sample of patients who participated. The questionnaire was created by the authors specifically for this purpose.

### Data Analysis

The analysis of all the information collected began with the literal transcription of the audio recordings. Interviews with service users lasted from 7 to 25 min. The analysis was then planned in three stages: discovery phase, analysis phase, and verification/interpretation phase (63).

In the discovery phase, we determined the parts on which the analysis would focus. The recorded interviews were transcribed literally. The researcher read each interview several times in order to identify the main topics.

In the analysis phase, all the information was coded and categorised using the qualitative research software Atlas.ti v.9. as the document manager.

In the verification/interpretation phase, the findings were contrasted with the objectives and broader theoretical frameworks. The validity of the analysis in qualitative research is present both in the systematic process of obtaining the data and in analysing these data (64). The authors of the study analysed the interviews and were in permanent telephone and e-mail contact with each other, conducting three meetings to discuss and agree on the information as a whole. After contrasting the differences with the available bibliography and with the conceptual framework itself, the first three co-authors refined and agreed on the most relevant data that gave meaning to the coding and analysis units. To ensure greater methodological triangulation, the results were presented to the remaining co-authors, who are clinical professionals in the field of mental health and participated in the selection of the subjects so they could corroborate the validity and credibility of the analysis performed. In addition, some of the subjects were informed of the research results during the regular follow-up visits. Extracts from the interviews have been included to illustrate the different topics.

## Results

### Elaboration of the Analysis Clusters

With the Two Step Method and depending on the variables (the presence of clinical symptoms of insomnia, the ISI scale ordinal variable result and the EQ-VAS scale continuous variable result) three analysis clusters were created from the total sample of 170 patients who presented at least one of the diagnostic criteria for insomnia. This procedure showed a good value in the cohesion and separation profile (0.6). The Two Step procedure was then contrasted with the elaboration of three hierarchical clusters using the Ward method. Cohen's Kappa statistic showed that the ratio between the two procedures was 0.542 (*p* = 0.000), thus demonstrating a moderate concordance between the two methods.

We did not obtain statistically significant differences between the sociodemographic variables in the three clusters and we did observe differences (*p* < 0.05) between the ISI and EQ-VAS variables (Supplementary Table 1).

### Socio-Demographic Characteristics of the Sample

The total sample of interviewees was 31 patients with a mean age of 51.26 (SD ± 13.302) and an age range of between 23 and 78 years. The distribution of the sample according to sex was 19 male patients (61.3%) and 12 female patients (38.7%). Most participants were single (61.3%; *n* = 19), had secondary education (54.8%; *n* = 17), had a recognised work disability (58.1%; *n* = 18) and their income level was generally below the minimum interprofessional wage (67.7%, *n* = 21). Regarding medication, 100% of the sample was treated with antipsychotic medication (*n* = 31), 54.8% took antidepressants (*n* = 54), 9.7% took mood stabilisers (*n* = 3) and 32.2% of all patients were prescribed anxiolytics (*n* = 10). The distribution of the sample according to the insomnia severity clusters and their sociodemographic characteristics can be seen in Table 1. This distribution does not show statistically significant differences between the three groups, except for the degree of disability (*p* = 0.041). However, this is not a relevant data item because any value above 33 already recognises a significant degree of disability in the affected person.

**Table 1:** Description of the sample of patients interviewed and the statistical significance.

	**Cluster 1—Severe-Moderate insomnia**		**Cluster 2—Mild insomnia**		**Cluster 3—No insomnia with symptoms**		**χ^2^/*t***	***p***
	***n***	**%**	***n***	**%**	***n***	**%**		
*Sex*							4.844	0.089
Male	8	88.9	5	41.7	6	60.0		
Female	1	11.1	7	58.3	4	40.0		
*Age (mean ± SD)*	53 (±10.83)		52 (±14.10)		49 (±15.25)		0.210	0.812
*Marital status*							7.528	0.275
Single	8	88.9	5	41.7	6	60.0		
Married/In relationship	0	0.0	4	33.3	2	20.0		
Separated/Divorced	0	0.0	2	16.7	2	20.0		
Widowed	1	11.1	1	8.3	0	0.0		
*Educational attainment*							7.600	0.269
No primary school	2	22.2	0	0.0	0	0.0		
Primary school	3	33.3	4	33.3	4	40.0		
Secondary school	4	44.4	8	66.7	5	50.0		
University	0	0.0	0	0.0	1	10.0		
*Employment status*							6.202	0.789
Special work centre	1	11.1	0	0.0	0	0.0		
Freelancer	1	11.1	0	0.0	0	0.0		
Salaried employee	0	0.0	1	8.3	1	10.0		
Unemployed	1	11.1	2	16.7	2	20.0		
Disability	5	55.6	7	58.3	6	60.0		
Retired	1	11.1	2	16.7	1	10.0		
*Degree of disability* (mean ± SD)	66 (±2.47)		64 (±4.66)		70 (±7.87)		3.655	0.041
*Link to resources*							4.377	0.626
No link	6	66.7	4	33.3	6	60.0		
CRS	2	22.2	4	33.3	2	20.0		
Pre-employment	1	11.1	1	8.3	1	10.0		
Social club	0	0.0	3	25.0	1	10.0		
*Income level*							4.876	0.560
No income	1	11.1	1	8.3	0	0.0		
Less than minimum wage	5	55.6	7	58.3	9	90.0		
Minimum wage	0	0.0	1	8.3	0	0.0		
More than minimum wage	3	33.3	3	25.0	1	10.0		
*BMI (mean ± SD)*	30.51 (±5.92)		28.00 (±4.68)		30.00 (±5.65)		0.658	0.526
*Antipsychotics*							–	–
Yes	9	100.0	12	100.0	10	100.0		
No	0	0.0	0	0.0	0	0.0		
*Antidepressant*							4.463	0.107
Yes	7	77.8	7	58.3	3	30.0		
No	2	22.2	5	41.7	7	70.0		
*Mood stabilizer*							0.047	0.977
Yes	1	11.1	1	8.3	1	10.0		
No	8	88.9	11	91.7	9	90.0		
*Anxiolytics*							0.695	0.706
Yes	2	22.2	4	33.3	4	40.0		
No	7	77.8	8	66.7	6	60.0		

### Result of the Hermeneutic Analysis

The analysis units that have emerged from the detailed reading of the interviews were organized into 3 categories and 16 subcategories. This made it possible to classify the 658 textual citations and respond to the experience of insomnia and its conditioning illness, sickness, and disease. The classification and definition of each category can be seen in Table 2. The results for each identified category are presented below.

**Table 2:** Coding.

**Category**	**Subcategory**	**Definition**
*Explanatory model*	Definition of insomnia	Own definition of what insomnia is
	Evolution	Modification of sleep and insomnia due to the passage of time
	Cause	Elements that cause insomnia
	Added difficulties	Aspects that worsen the prognosis, sometimes not depending on the actions of the person themselves
	Clinical	Signs and symptoms of insomnia
	Consequences	Consequences caused by insomnia
	Objectives / Expectations	What they would like to achieve regarding sleep. The sense of sleep
*Personal strategies*	Own solutions	Solutions tried individually without prior indication
	Daily activities	Performing tasks or activities that improve sleep
	Involvement	Patient's attitude towards the pathology and its treatment
	Verbalization	Voluntary act of communicating the presence of a problem to health professionals
*Health response*	Clinical care	Response of health professionals to patients' demands
	Pharmacological treatment	Pharmacological treatment that follows and perception of this
	Advice	Advice provided by health professionals
	Psychoeducational group	Opinions on group interventions for treating insomnia
	Desired treatment	The treatment that patients would like to receive without knowing the options

#### The Explanatory Model of Insomnia

The patients who participated in the interviews clearly defined the different characteristics of insomnia, their own experience and even the ideal of sleep they would like to enjoy. This explanatory model allows us to understand how their insomnia affects their life reality and what clinical strategies can be used to help improve the disorder they suffer from.

The concept that the patients repeated most was that insomnia is like a disease that prevents them from sleeping and resting, generating a feeling of constant anxiety:

“*Being in bed and starting to think… then falling asleep… waking up and sleeping, waking up and sleeping, the night is not over yet, the night is not over. Opening your eyes, closing your eyes… being like that all night, unable to fall asleep.”* (PAC31.1)

“*It's a disease, right? Like they say. It's a disease that prevents you from sleeping and you have a really bad time.”* (PAC4.2)

In most patients, the suffering they describe began many years ago, especially in those in the cluster with severe/moderate insomnia, and they describe this life experience clearly. Patients in the mild insomnia cluster also describe the problem with a clear perception of its evolution but in less detail. However, the patients in the cluster *no insomnia with symptoms*, do not have this evolutionary perception of the sleep problem. Some of the descriptions of life situations perceived as triggers for the sleep problem include: military service, the onset of the mental disorder itself or even the death of a family member. These traumatic events lead to the sleep problem becoming worse as time goes on, which has also been described by the interviewees.

Regarding the causes identified by patients, we found two aspects that were repeated in most interviews: poor sleep hygiene involving behaviours that increase the chronic manifestation of the problem, and repetitive ruminations and thoughts, some of these being obsessive and pervading their minds when they try to fall asleep. Ruminations are present in all three clusters, regardless of the severity of their insomnia, and are the characteristic that causes the participants the most anxiety and distress.

“*When I have these thoughts, so, so many that… it's that thoughts come to me. I also jump from one thought to another, from one thought to another. Lately, the smallest things that happen during the day, I go over them at night, the slightest thing, and they go round and round in my head, and even though I say to myself that I don't want to think about it, I want to have a clear mind, it makes no difference, the thoughts just keep coming back, over and over again, and I jump from one thought to another…. I jump from one thought to another.”* (PAC14.1)

“*Yes! Of course, because I want to sleep soundly without waking up and this problem comes to me, what I want is to get it out of my head so I can keep sleeping, I don't want to think about this problem anymore, I just want to keep sleeping.”* (PAC8.2)

“*Social problems, family problems, sometimes if I have a problem it's difficult for me to fall asleep because I can't get it out of my head and I can't disconnect and then it's even more difficult for me to fall asleep.”* (PAC26.3)

Other aspects of the underlying schizophrenic disorder itself, such as delusional ideas, comorbidities of physical and/or organic diseases, or problems with urinary containment, further aggravate the causes of insomnia.

We detected differences among our sample clusters regarding the persistence of this. Patients with severe/moderate insomnia, in particular, and patients with mild insomnia, are greatly impacted. In contrast, in patients without insomnia, but with the presence of at least one of the diagnostic symptoms, the frequency and duration of problems with falling asleep and staying asleep are less intense and they have a perception of being less affected; some of the respondents in this group even believe they do not have any clinical symptoms.

“*I hadn't changed it before… but then… I started to change my routine, I started to change it radically and then I couldn't sleep at night and during the day I was awake. Then I sleep during the day, because seeing as I don't sleep at night, I sleep during the day.”* (PAC17.1)

“*At first when it's time to go to bed… I'm very sleepy but when I get into bed then I can't sleep. Maybe I fall asleep at 2 o'clock in the morning.”* (PAC3.2)

“*I feel calm, I don't get anxious, but I don't fall asleep sometimes… but in the end if I wait and keep calm I fall asleep. I don't get frustrated.”* (PAC5.3)

All these symptoms and nocturnal suffering have many repercussions during the day. The patients described a long list of consequences: feeling down with no energy, feeing nervous and anxious, restlessness, a lack of motivation, difficulties concentrating and in performance, memory problems, bad moods, difficulty getting up, irritability, apathy, restlessness, and even increased rumination and worsening of the underlying disorder. This significantly limits their ability to perform daily tasks and activities and clearly affects their quality of life.

“*What worries me most is that my quality of life is not the same. It worsens my quality of life and it aggravates the symptoms of my illness. Of course the brain needs to rest, but if I already have… a problem then you need to add to that the fact that I spend all night turning things over in my head, I don't know what, I don't know how many, this and that, of course, and then another day arrives and I feel disorientated because I don't even know what day it is… do you know what I mean? … my insomnia worsens my quality of life*.” (PAC25.2)

However, if there is one aspect in which virtually all participants, even patients in the cluster without insomnia with symptoms, agreed on it is what they would like to experience regarding sleep, and that is rest. To have the feeling of resting physically and mentally, as they are aware of the importance of this in their daily lives.

“*It's just as important as eating. If you don't eat you don't live. If you don't sleep you don't live.”* (PAC11.3)

“*Sleep is a time of relaxation to escape from everyday life and rest and feel better the next day.”* (PAC12.2)

“*Rest, rest, mainly, rest.”* (PAC14.1)

#### Personal Strategies for Facing Insomnia

Faced with all this discomfort, the patients developed a series of strategies to try to deal with their insomnia themselves.

The strategies that patients use to fall asleep are mostly behavioural, such as putting on music or the radio, drinking a cup of herbal tea, following sleep hygiene guidelines, even performing relaxation techniques and cognitive strategies in which the patient consciously tries to mentally stop the ruminations and thoughts that prevent them from falling asleep. The participants find that most of these acts are usually ineffective, except in certain cases when certain relaxation techniques, prior training by clinicians, or a routine based on sleep hygiene, have been found to be effective.

“*Sometimes, maybe, I do a relaxation exercise before bed. When I had the anxiety crisis of more… more… sometimes like this… in the hospital I was given a CD with relaxation exercises by a nurse and then sometimes I put it on before I go to bed. I try to put it on to do these exercises*.” (PAC8.2)

“*If I eat earlier my stomach is calm and I sleep better.”* (PAC26.3)

However, there are other aspects, such as the activities carried out during the day, that have clear repercussions and a subjective benefit for the improvement of sleep, both for falling asleep and for staying asleep. Staying active, following routines during the day, working and doing exercise, and consequently being tired, are the activities that bring most satisfaction when it comes to going to sleep. This is expressed more by groups *no insomnia with sympto*ms and with *mild insomnia*. However, the patients in the group with severe /moderate insomnia are less aware of this need and almost never express it.

“*The only thing for me is to be empty from not doing anything during the day… I feel like going to bed. Then when I'm in bed. It makes me sleep.”* (PAC17.1)

“*The fact of having had a good day, full of exercise, like… as they say… to have exercised your mind, your body, your mind, well, it's very important in order to sleep well. You do notice the difference.”* (PAC4.2)

Nevertheless, it depends on the patients' commitment and motivation whether they end up applying these strategies, or at least attempting them. It is the patients with severe/moderate insomnia who speak of this lack of motivation and commitment to self-improvement, despite being those who suffer most and despite possessing information and knowledge about strategies that could lead to a clear improvement in sleep.

The communicative capacity of the patients themselves also affects their ability to express their sleep problems and their search for more knowledge. Many of the interviewees did not immediately describe these problems, not believing it necessary to express them or not initially appreciating that they were serious enough. Therefore, they have continued to adopt compensatory strategies that may have made the problem worse over time. Some patients also reported that they were afraid to explain these problems in case the therapist thought that, rather than a sleep problem, it was the patient's underlying pathology.

“*It's not because I feel ashamed, I don't care, If I have to tell I just have to, but I think that people, well… as it's not such a serious problem… because it doesn't matter how important it is but when you have it over time, it starts to take its toll.”* (PAC25.2)

“*No, I don't want them to think I am crazier than I am.”* (PAC1.1)

#### The Healthcare Response to Insomnia

The patients' suffering and the failure of their individual strategies means that they required a clinical response to their sleep problems, which they experience in different ways depending on whether it is aimed at preventing or treating insomnia.

The clinical care that patients receive is strongly linked to a pharmacological response, but the questions that are asked to determine their state of sleep are simple questions that do not analyse in depth the real problems that patients themselves experience. Moreover, such questioning is not common in most follow-up visits and the care provided varies greatly depending on the professional assigned to each patient.

“*Don't ask too much […] Everything is very pharmacological.”* (PAC19.2)

“*I don't think so, because there are a lot of people who have it and it's like… if you don't sleep well… take a pill, take one of those… but I think they don't really get involved, the doctors, they don't really get involved… they're not really sleep specialists… I'm speaking in general, about GPs, they don't put themselves in the shoes of the person who is sleeping badly,…it's really terrible… it's…”* (PAC21.1)

“*I think sp; I haven't seen the nurse in a long time but she always asks me how I've been sleping.”* (PAC29.3)

It is this pharmacological response that patients associate with effective improvement, as it aims to stop repetitive thoughts quickly, and leads them to believe that they will be able to sleep according to their expectations. This type of treatment is the most common solution offered to patients. Although they perceive this response as powerful and effective, in many cases they see it as a solution that has had little thought put into it and one that has unwanted secondary effects, such as drowsiness in the morning, addiction or tolerance to pharmacological drugs. Some patients also state that they are already taking a lot of medication because of their disorder and are reluctant to increase this.

“*Some pills let me… they would put a horse to sleep because… otherwise I can't explain how I can take so long to get to sleep.”* (PAC28.1)

“*It doesn't affect me earlier on, but later when I've already fallen asleep… it seems that the next day I'm really tired, really sleepy, so I decided to stop taking them.”* (PAC15.1)

In contrast, advice on habits, sleep hygiene, and relaxation techniques is favourably accepted. These are good solutions in some cases, but we observed that when the patient expresses their problems, they feel more desperate when these measures seem to be ineffective. However, few professionals choose to tell patients about these types of strategies and the patients themselves show little hope or interest.

“*When I was last admitted to hospital I was taught to meditate and relax without medication, in the hospital there was a nurse who was really nice and she brought us all together, whoever wanted to relax and she explained a breathing technique and.. a mind control technique, of seeing beautiful things and some very relaxing music and that helped me sleep and I kept doing it. She is the only person who has told me about something other than drugs.”* (PAC26.3)

“*Yes, they gave me (advice) but I still have insomnia anyway*.” (PAC20.2)

“*Well, I don't remember, but it seems to me like nothing more than pills. I don't think they gave me any advice.”* (PAC10.2)

A third type of health response is group intervention, which despite being an instrument that some patients value positively as a resource for more information and knowledge, most do not recognise it as useful. They are reluctant and disbelieving of the clinical possibilities of these groups. However, when the results offered by this type of intervention and the cognitive-behavioural activities and techniques involved are explained to the patients, they are more willing to participate.

“*Because it's just talking and talking and we don't learn anything from each other. Each person has their own opinion…they talk a lot but they don't believe much*.

***And if the group was not for giving your opinion, but rather for following some guidelines presented by a professional? (asks the interviewer)***

*Well, that sounds a bit… it sounds a bit better. Yes. I believe more. Exactly, I believe more in that. Yes, I believe. This would be better! Yes! I believe much more in that than in the other type of group.”* (PAC14.1)

What is evident in the interviews is the patients' willingness to be treated holistically, without giving up any of the possible strategies, if in this way they can achieve their goal of clearing their minds and resting. Patients are looking for serious, powerful and effective treatments.

## Discussion

This article is based on the narratives of patients with schizophrenia regarding their experiences of insomnia, their responses to their sleep problem, and the responses of healthcare professionals with clinical resources. This field of knowledge, as described by Martínez-Hernáez and Correa-Urquiza (65), allows us to interpret how the actual disorder and suffering is experienced from the site of the affliction, making it possible to reveal knowledge that exists but which is constantly denied, hidden and neglected.

The explanatory model of insomnia described by the patients interviewed agrees with the Behavioural Model of Insomnia proposed by Spielman et al. (66) in 1987, which describes chronic insomnia as the sum of three factors: predisposing factors, precipitating factors and perpetuating factors. In the narratives, patients verbalise these three factors, giving evidence of life situations that have precipitated and perpetuated their insomnia. Schizophrenia is already in itself a predisposing factor (20, 67) and some of the clinical features have been observed as precipitating factors.

One of the most important problems that participants in the study have described as a cause is the presence of invasive and ruminative thoughts during the process of falling asleep. This feature has already been described by Morrison and Baker (68) as common in patients with schizophrenic disorder, and is more present in patients with insomnia (43). According to Luca (69), the poorly adaptive rumination that some patients show and which negatively influences falling asleep may be evidence of a cognitive vulnerability and a dysfunctional coping strategy of the psychosis itself and can also be associated with positive and negative symptoms of schizophrenia (70). Chiu et al. (43) concluded that psychological sleep interventions should address voices and other psychotic symptoms if these are not well-controlled by the antipsychotic treatments.

Patients suffering from chronic insomnia not only have problems that affect health (12), but also problems that affect their social and occupational functioning. They frequently complain of emotional, cognitive and behavioural symptoms and deterioration in social and occupational areas, issues that lead to increased absenteeism (71, 72). The repercussions and consequences observed in our sample, such as apathy, lack of concentration and daytime sleepiness, are consistent with those described by Faulkner and Bee (39) in their qualitative analysis of a sample of patients with schizophrenia.

Another consequence that the informants described is a lack of motivation and willingness to perform activities due to insomnia. A connexion to the community or work resources improves the prognosis of patients with a mental disorder (73), which is hampered by the effect of insomnia on the performance of everyday activities and quality of life (13). Some of the patients have reported that starting a job or training has substantially improved their sleep; however, the lack of activity presented by many of the patients (74) and the high presence of sedentary activities also lead to lower sleep pressure and the alteration of the homeostatic cycle.

Communicating and verbalising sleep problems is an important factor in correctly approaching the disorder; however, patients expressed, in several of the interviews, that they have difficulty communicating their problems and feelings to professionals. Engin et al. (75) observed in their study on anger and alexithymia in patients diagnosed with insomnia that these patients have greater difficulty in expressing their feelings than the group that did not have insomnia. This is a factor to keep in mind when collecting an anamnesis and deciding on the correct approach. In their narratives, the patients often said that they frequently do not explain their sleep problems partly due to the difficulties they have in communicating and expressing themselves.

The non-communication of these disorders for fear that professionals might confuse the symptom with a re-exacerbation of their underlying disorder was also described by some of the patients in our sample and was similarly observed by Chiu et al. (45). Some of the interviewees in our study expressed that the therapeutic link and perception of empathy with health professionals, an aspect widely studied in patients with schizophrenia (76, 77), is an important factor in improving their ability to communicate and express their sleep problems.

Sleep disorders and insomnia are usually treated with pharmacological drugs, although evidence shows that cognitive-behavioural and psychoeducational interventions are more effective long-term treatments, and even reduce the economic cost (78). Cognitive-behavioural therapy has shown statistically significant results in the treatment of insomnia (79) and in treating patients with comorbidities of both mental and organic diseases (80). Cheung et al. (34) propose combining the two treatments simultaneously to take advantage of their competitive values, an aspect supported by the narratives in our sample.

The perceptions of our patients regarding treatments for insomnia agree with the description made by Davy et al. (35) in their analysis. Those authors found that patients perceive that health care professionals offer interventions that are too simplistic for their health problem. Patients, for the most part, believe that professionals care, but that their solutions do not go beyond pharmacological treatments. These pharmacological treatments are also experienced in contradictory ways by the patients, as described by Waters et al. (38). In our sample, the difference is also very clear between patients who consider that a pharmacological treatment is the right option and others who state that it is just the easy answer.

What is clear in the sample studied is that patients do not know what is the optimal treatment for insomnia. There is ample evidence that cognitive-behavioural therapy is effective in treating sleep disorders and is the technique of choice for chronic insomnia ahead of pharmacological treatment (81) because it reduces symptoms by 50% during the treatment phase and remains beneficial after 12 months (82, 83). It is also the therapy of choice for treating insomnia according to the *European Guideline for the Diagnosis and Treatment of Insomnia* (84). However, despite this evidence, due to their unfamiliarity with the treatment, informants doubt the veracity and potential of this therapy, which was also observed in the study by Messari and Hallam (85) in patients with psychosis.

Nevertheless, even short, single-session interventions (86) can significantly improve sleep-related worries, reduce dysfunctional sleep beliefs, and improve subjective perception. This is why the intervention of clinical professionals in patient follow-up visits can lead to important changes in the experience of this disorder.

### Limitations of the Study

Our study has some limitations. This is a cross-sectional study with a sample of patients from a single mental health centre and the analysis only corresponds to the population of this centre with this pathology. Nevertheless, the study adequately describes each procedure so that they can be reproduced in other centres and has been performed using a methodology similar to other studies. Another limitation is that the participants were not selected randomly, and only those patients who were open to being audio-recorded participated; therefore, introducing a possible bias. In addition, the fact that the interviews were conducted and recorded by one of the therapists at the same centre may have had some influence on the participants' responses. Another limitation in our study is the short duration of some of the conducted interviews, in part, due to poor and spontaneous communication, a feature already described before in population with schizophrenic disorder (87–89), but no less significant and interesting. A comparison has not been made between the different clusters, as it was not the aim of our study and, we did not focus our analysis on studying the bidirectionality (article) between insomnia and mental disorders, but on understanding the experience of insomnia itself. Finally, another limitation of this investigation is we did not use the diagnostic criteria of the newest diagnostic manuals, such as the DSM-V or the ICSD-3, because of the lack of validated diagnostic questionnaires for this purpose. The OSQ was finally selected for the insomnia diagnose and not as clinical interview.

However, this study also has some strong points that should be highlighted. First, this study qualitatively analyses the experience of insomnia in patients with schizophrenic disorder from an outpatient mental health centre allying the explanatory model, personal resources and healthcare response, thus ensuring the clinical variability of informants in relation to the severity of their insomnia and their perception of their quality of life through stratification by analysis clusters. Secondly, the sample (*n* = 31) allowed us to reach the saturation of concepts necessary to be able to obtain consistent results and a subsequent analysis. Thirdly, in addition to the methodological procedure, verifying the findings with the informants themselves and with the professionals who recruited the patients has ensured a correct triangulation of the information. Finally, we believe it is important to note that this is the first study in Spain that researches the experience of patients with schizophrenia who suffer from insomnia.

### Future Implications

Finding out how people experience and perceive insomnia, how it affects their quality of life and how they perceive the clinical care they receive will enable treatments to be focused more effectively. It is important to be more exhaustive during the anamnesis, looking for insomnia-related problems, letting patients express themselves properly, encouraging greater involvement and motivation among patients, giving better explanations regarding therapeutic strategies and, most of all, assessing if ruminations or intrusive thoughts are clinical symptoms of an acute manifestation of the underlying disorder. All these considerations should be reflected in the design of future cognitive-behavioural and psychoeducational interventions specific to these patients. Also, when interventions are designed, there should be an emphasis on linking patients with community, social, training or employment resources, if possible.

### Future Lines of Research

In our research, we have studied the insomnia experiences of patients with a schizophrenic disorder. In future studies, it would be interesting to compare their experiences with people who do not suffer schizophrenia and carry out a comparative study regarding the severity of the symptoms, thus allowing use to plan care strategies according to the population characteristics.

## Conclusions

Insomnia is a sleep disorder experienced by the study participants as a health problem that conditions them on both the physical and mental levels. It has a large impact that leads to emotional discomfort. The informants' main desire is to achieve a mental break that allows them to rest and escape from reality. The inability to sleep properly promotes the appearance of repetitive thoughts, feelings of guilt and anxiety, and daytime dysfunction. It is important that health professionals intervene preventively to stop the disorder from becoming chronic. Patients with severe/moderate and mild insomnia require and request powerful and effective interventions such as cognitive-behavioural therapy for their disorder. Conversely, patients without insomnia (with the presence of one or more diagnostic symptoms) need preventive and psychoeducational treatment. The quality of life of people with schizophrenic disorder and insomnia would be improved by offering strategies that combine the patient's own perspective and experience with encouragement and motivation.

## Data Availability Statement

The raw data supporting the conclusions of this article will be made available by the authors, without undue reservation.

## Ethics Statement

The studies involving human participants were reviewed and approved by Comitè d'Ètica d'Investigació amb medicaments (CEIm) Fundació Unió Carrer Valencia, 333 baixos 08009, Barcelona, Spain. The patients/participants provided their written informed consent to participate in this study.

## Author Contributions

DB-M, M-AM-P, and AB-E: conceptualization and data curation. DB-M: formal analysis and writing—original draft. DB-M, EM, AD-C, MM, and JV: investigation. DB-M, AB-E, and M-AM-P: methodology. M-AM-P and AB-E: supervision. DB-M, AB-E, EM, MM, JV, AD-C, ML-R, and M-AM-P: writing—review and editing. All authors have read and agreed to the published version of the manuscript.

## Funding

The article-processing charge was defrayed by a grant from the Col·legi Oficial d'Infermeres i Infermers de Barcelona (COIB) to support the publication of articles in open-access scientific journals.

## Conflict of Interest

The authors declare that the research was conducted in the absence of any commercial or financial relationships that could be construed as a potential conflict of interest.

## Publisher's Note

All claims expressed in this article are solely those of the authors and do not necessarily represent those of their affiliated organizations, or those of the publisher, the editors and the reviewers. Any product that may be evaluated in this article, or claim that may be made by its manufacturer, is not guaranteed or endorsed by the publisher.
