# Supplementary figures and images for: The Experience of Insomnia in Patients With Schizophrenic Disorder: A Qualitative Study
Source: Front Psychiatry. 2022 Jan 17;12:805601. doi: 10.3389/fpsyt.2021.805601 (PMC8801919; doi:10.3389/fpsyt.2021.805601)

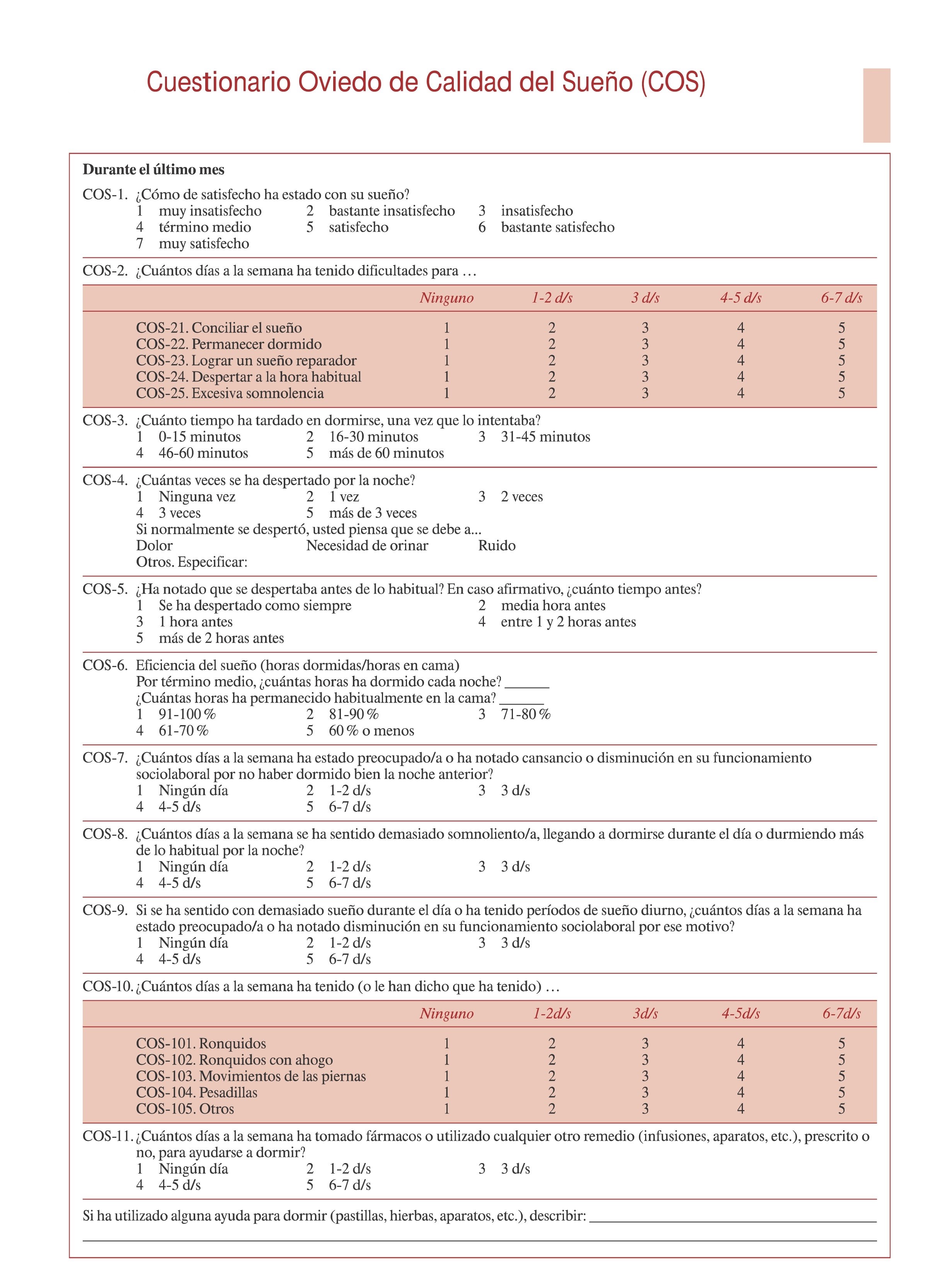

Supplement: Supplementary file 1 [file Image_1.JPEG]
