# Supplementary material for: The Experience of Insomnia in Patients With Schizophrenic Disorder: A Qualitative Study
Source: Front Psychiatry. 2022 Jan 17;12:805601. doi: 10.3389/fpsyt.2021.805601 (PMC8801919; doi:10.3389/fpsyt.2021.805601)
Supplement: Supplementary file 2 [file Table_1.docx]

**Supplementary Table 1** - Socio-demographic characteristic clusters, Two-Step procedure and statistical significance

|  | Cluster 1 – Severe-Moderate Insomnia | | Cluster 2 – Mild Insomnia | | Cluster 3 – No insomnia with symptoms | | χ² / *t* | *p* |
| --- | --- | --- | --- | --- | --- | --- | --- | --- |
|  | n | % | n | % | n | % |  |  |
| *Sex* |  |  |  |  |  |  | 0.604 | 0.739 |
| Male | 20 | 55.6 | 37 | 56.9 | 43 | 62.3 |  |  |
| Female | 16 | 44.4 | 28 | 43.1 | 26 | 37.7 |  |  |
| *Age (mean ± SD)* | 50 (*±*10.58) | | 50 (*±*12.00) | | 51 (*±*12.64) | | 0.065 | 0.937 |
| *Marital status* |  |  |  |  |  |  | 4.798 | 0.570 |
| Single | 20 | 55.6 | 30 | 46.2 | 37 | 53.6 |  |  |
| Married / In relationship | 8 | 22.2 | 21 | 32.3 | 16 | 23.2 |  |  |
| Separated / Divorced | 7 | 19.4 | 10 | 15.4 | 15 | 21.7 |  |  |
| Widowed | 1 | 2.8 | 4 | 6.2 | 1 | 1.4 |  |  |
| *Educational attainment* |  |  |  |  |  |  | 7.984 | 0.239 |
| No primary school | 6 | 16.7 | 7 | 10.8 | 4 | 5.8 |  |  |
| Primary school | 19 | 52.8 | 31 | 47.7 | 35 | 50.7 |  |  |
| Secondary school | 8 | 22.2 | 26 | 40.0 | 27 | 39.1 |  |  |
| University | 3 | 8.3 | 1 | 1.5 | 3 | 4.3 |  |  |
| *Employment status* |  |  |  |  |  |  | 11.906 | 0.291 |
| Special work centre | 2 | 5.6 | 3 | 4.6 | 2 | 2.9 |  |  |
| Freelancer | 2 | 5.6 | 0 | 0.0 | 0 | 0.0 |  |  |
| Salaried employee | 1 | 2.8 | 9 | 13.8 | 7 | 10.1 |  |  |
| Unemployed | 2 | 5.6 | 7 | 10.8 | 7 | 10.1 |  |  |
| Disability | 25 | 69.4 | 40 | 61.5 | 45 | 65.2 |  |  |
| Retired | 4 | 11.1 | 6 | 9.2 | 8 | 11.6 |  |  |
| *Degree of disability* (mean ± SD) | 63 (*±*15.92) | | 54 (*±*26.14) | | 56 (*±*22.77) | | 2.157 | 0.119 |
| *Link to resources* |  |  |  |  |  |  | 1.719 | 0.944 |
| No link | 25 | 69.4 | 43 | 66.2 | 43 | 62.3 |  |  |
| CRS* | 5 | 13.9 | 13 | 20.0 | 12 | 17.4 |  |  |
| Pre-employment | 2 | 5.6 | 2 | 3.1 | 4 | 5.8 |  |  |
| Social club | 4 | 11.1 | 7 | 10.8 | 10 | 14.5 |  |  |
| *Income level* |  | |  | |  |  | 4.555 | 0.602 |
| No income | 4 | 11.1 | 4 | 6.2 | 10 | 14.5 |  |  |
| Less than minimum wage | 21 | 58.3 | 36 | 55.4 | 40 | 58.0 |  |  |
| Minimum wage | 2 | 5.6 | 2 | 3.1 | 3 | 4.3 |  |  |
| More than minimum wage | 9 | 25.0 | 23 | 35.4 | 16 | 23.2 |  |  |
| *BMI (mean ± SD)* | 30.64 (*±*5.81) | | 29.46 (*±*5.62) | | 28.91 (*±*5.87) | | 1.065 | 0.347 |
| *Insomnia Severity Index* |  | |  | |  | | 340.000 | 0.000 |
| No Insomnia | 0 | 0 | 0 | 0.0 | 69 | 100.0 |  |  |
| Mild Insomnia | 0 | 0 | 65 | 100.0 | 0 | 0.0 |  |  |
| Moderate Insomnia | 28 | 71.5 | 0 | 0.0 | 0 | 0.0 |  |  |
| Severe Insomnia | 8 | 28.5 | 0 | 0.0 | 0 | 0.0 |  |  |
| *EQ-VAS* *(mean ± SD)* | 48.9 (*±*18.94) | | 57.9 (*±*18.63) | | 69.5 (*±*16.11) | | 17.395 | 0.000 |
| *Antipsychotics* |  |  |  |  |  |  | 1.625 | 0.444 |
| Yes | 36 | 100.0 | 64 | 98.5 | 69 | 100.0 |  |  |
| No | 0 | 0.0 | 1 | 1.5 | 0 | 0.0 |  |  |
| *Antidepressant* |  |  |  |  |  |  | 5.685 | 0.058 |
| Yes | 19 | 52.8 | 20 | 30.8 | 22 | 31.9 |  |  |
| No | 17 | 47.2 | 45 | 69.2 | 47 | 68.1 |  |  |
| *Mood stabilizer* |  |  |  |  |  |  | 2.581 | 0.275 |
| Yes | 2 | 5.6 | 4 | 6.2 | 9 | 13.0 |  |  |
| No | 34 | 94.4 | 61 | 93.8 | 60 | 87.0 |  |  |
| *Anxiolytics* |  |  |  |  |  |  | 0.786 | 0.675 |
| Yes | 19 | 52.8 | 37 | 56.9 | 34 | 49.3 |  |  |
| No | 17 | 47.2 | 28 | 43.1 | 35 | 50.7 |  |  |

CRS: community rehabilitation service
